# 

**Published:** 1906-01-13

**Authors:** 


### NOTICE TO CORRESPONDENTS.

All MSS., Letters, Books for Review, and other matters intended for the Editor should be addressed The Editor, "The Hospital," The Hospital Buildings, 28 & 29 Southampton Street, Strand, W.C.

The Editor cannot undertake to return rejected MS., even when accompanied by stamped directed envelope.

**Notice to Advertisers and Subscribers.**

All Advertisements, Orders for Copies of The Hospital, and Business Communications should be addressed to **THE MANAGER (Not to the Editor),** The Hospital, 28 & 29 Southampton Street, Strand London, W.C.

The Cost of Subscription, post free, is as follows:—Per week, 3½d.; per quarter, 3s. 3d.; per half-year, 6s. 6d.; per annum, 13s. Foreign and Colonial:—Per annum, 19s. 6d., payable, in all cases, in advance.

**NOTICE.—Vol. XXXVIII. of "The Hospital" April to September 1905), in handsome cloth binding, will shortly be on sale at the office of this Journal, price 10s. 6d. Cases for binding the Numbers contained in this Volume 2s. Index to Vol. XXXVIII., 3½d., post free.**

**The Monthly Part for January is now ready. Price 1s., by post, 1s. 3d.**

### BOOKS RECEIVED.

E. Arnold.

"Recent Advances in Physiology and Bio-Chemistry." Edited by Leonard Hill, M.D.

T. B. Browne, Ltd.

"Advertisers' A.B.C., 1906."

J. and A. Churchill.

"The Management of a Nerve Patient." By A. T. Schofield, M.D.

Archibald Constable and Co.

"Lectures on Tropical Diseases." By Sir Patrick Manson.

Scientific Press, Ltd.

"Simple Advice to a Young Mother." By E. H. Young, C.M.B.

### Medical Appointments and Vacancies.

### APPOINTMENTS.

Benett, A. M., M.R.C.S.Eng., L.R.C.P.Lond., House Physician, General Hospital, Northampton.

Joy, G. P., M.A., Cantab.M.B., Ch.B.Edin., Assistant House Surgeon, General Hospital, Northampton.

### VACANCIES.

Birmingham and Midland Ear and Throat Hospital, Edmund Street.—House Surgeon.

Burnley, County Borough of.—Medical Officer of Health.

Chester General Infirmary.—House Surgeon.

Great Yarmouth Hospital.—House Surgeon.

Hospital for Consumption and Diseases of the Chest, Brompton.—Resident House Physicians.

Napsbury, near St. Albans, Herts, Middlesex County Asylum.—Assistant Medical Officer.

Northampton General Hospital.—House Surgeon.

Royal Free Hospital, Gray's Inn Road, W.C.—Medical Registrar; also a Surgical Registrar (females).

Salford Royal Hospital.—Honorary Assistant Surgeon.

University of London.—Examiners. One each in Medicine, in Surgery, and in Pathology.

York Dispensary.—Resident Medical Officer.

### THE NURSE'S BOOKSHELF

**HOW TO BECOME A NURSE:**
**How and Where to Train.**
Edited by Sir HENRY BURDETT, K.C.B.
A complete guide to the Nursing Profession.
2/- net, 2/4 post free.

**The Duties of a Nurse Explained.**
**NURSING HINTS TO PROBATIONERS ON PRACTICAL WORK.**
By M. ANNESLEY VOYSEY.
2/- net, 2/3 post free.
The *Lancet* says: "This is a very good practical book. . . . It contains the whole duty of Nurses given in plain language."

**PRACTICAL GUIDE TO SURGICAL BANDAGING AND DRESSINGS.**
By WM. JOHNSON SMITH, F.R.C.S.
Suitable size for Apron Pocket. 2/- post free.

**THE NURSE'S PRONOUNCING DICTIONARY OF MEDICAL TERMS AND NURSING TREATMENT.**
Compiled by HONNOR MORTEN.
New Edition edited by MARY I. BURDETT.
Suitable size for apron pocket, 2/- post free.

**OPHTHALMIC NURSING.**
By SYDNEY STEPHENSON, M.B., F.R.C.S.
New and Revised Edition. 3/6 net, 3/10 post free.

**THE MODERN NURSING OF CONSUMPTION.**
By Dr. JANE WALKER.
1/- net, 1/2 post free.

**SURGICAL WARD-WORK AND NURSING.**
By ALEX. MILES, M.D.
Illustrated. Cloth, 3/6 net, 3/10 post free.

**HOSPITAL SISTERS AND THEIR DUTIES.**
By EVA C. E. LÜCKES, Matron, London Hospital.
Third Edition, 2/6 post free.

**SIMPLE SANITATION:**
**The Practical Application of the Laws of Health to Small Dwellings.**
By M. LOANE. Cloth 1/- net, 1/2 post free.

**THE EXAMINATION OF THE URINE.**
By J. K. WATSON, M.D.
Cloth, 1/- net, 1/1 post free.

### THE BURDETT SERIES.

Bound in Cloth Boards, 1/- each, post free.

**PRACTICAL HINTS ON DISTRICT NURSING.**
By AMY HUGHES, General Superintendent, Queen Victoria's Jubilee Nurses.

**HOSPITAL AND PRIVATE NURSING.**
By S. S. ORME.

**THE MIDWIVES' POCKET BOOK.**
By HONNOR MORTEN.

**CARE OF CONSUMPTIVES.**
By Dr. W. H. DAW.

**FEVERS AND INFECTIOUS DISEASES.**
By Dr. WILLIAM HARDING.

**HOME NURSING OF SICK CHILDREN.**
By Dr. J. D. E. MORTIMER.

**MENTAL NURSING.**
By Dr. WILLIAM HARDING, Superintendent Berry Wood Asylum, Northampton.

**SICK NURSING AT HOME.**
By G. MOBERLY.

**GYNÆCOLOGICAL NURSING.**
By Dr. HAWKINS AMBLER.

**SYLLABUS OF LECTURES TO NURSES.**
By Dr. ANDREW DAVIDSON.

*Complete Catalogue of Nursing Manuals, Case Books, Charts, &c., post free on application.*

### THE SCIENTIFIC PRESS, LTD.,
**28 & 29 Southampton Street, Strand, LONDON.**

